# Association Between miRNAs and the Diagnosis, Prognosis, and Recurrence of Patients with Meningioma: A Systematic Review

**DOI:** 10.1007/s10571-026-01665-2

**Published:** 2026-02-07

**Authors:** Daniel Alfonso Nieva Posso, Daniel Andrés Nieva-Posso, Carlos Arturo González-Acosta, Diego Alejandro Vargas, Herney Andrés García-Perdomo, Lina V. Becerra-Hernández, Efraín Buriticá-Ramírez

**Affiliations:** 1 Centro de Estudios Cerebrales, Facultad de Salud, Universidad del Valle, Calle 4B # 36-00, Cali, 760032 Colombia; 2 UROGIV Research Group, School of Medicine, Universidad del Valle, Calle 4B # 36-00, Cali, 760032 Colombia; 3 Pathology Unit, Clínica Imbanaco Grupo QuirónSalud, Calle 4B # 36-00, Cali, 760032 Colombia; 4 Division of Urology/Uro-Oncology, Department of Surgery, School of Medicine, Universidad del Valle, Calle 4B # 36-00, Cali, 760032 Colombia; 5 Basic Health Sciences Department, Pontificia Universidad Javeriana, Calle 4B # 36-00, Cali, Colombia

**Keywords:** Meningioma, MicroRNA, Diagnosis, Prognosis, Recurrence

## Abstract

To evaluate the diagnostic, prognostic, and recurrence-related roles of microRNAs (miRNAs) in meningioma based on expression profiles across different biological samples. This systematic review was conducted in accordance with Cochrane Collaboration and PRISMA guidelines. A search was performed in PubMed, Scopus, Web of Science, and Google Scholar without language restrictions. Cohort and case–control studies assessing miRNA expression in tumor tissue, serum, or cerebrospinal fluid from patients with histologically confirmed meningioma were included. Risk of bias was evaluated using the Newcastle–Ottawa Scale. Bioinformatic pathway enrichment analyses were conducted for recurrently miRNAs. Twenty-three studies involving 1615 participants were included, of whom 1461 had meningiomas (WHO grades 1–3). Overall, 153 miRNAs were evaluated, including 90 upregulated, 45 downregulated, and 18 without significant differential expression. miR-21, miR-34a, and members of the miR-181 family were consistently associated with tumor grade and progression. Tumor recurrence was most frequently linked to miR-224-5p, miR-331-5p, miR-29c-3p, and miR-219-5p. Circulating miRNAs, particularly miR-497 and miR-219, demonstrated diagnostic potential in serum and cerebrospinal fluid. Pathway analyses highlighted enrichment in metabolic and signaling pathways, including sphingolipid, sulfur, and fatty acid metabolism. This review identifies miR-21, miR-224-5p, miR-331-5p, miR-29c-3p, and circulating miR-497 and miR-219 as the most promising miRNA biomarkers for meningioma diagnosis, grading, and recurrence monitoring. Despite methodological heterogeneity, the consistency of these findings across independent studies supports their translational potential. Nonetheless, large, standardized studies with independent validation cohorts are required prior to clinical implementation.

**Supplementary Information:**

The online version contains supplementary material available at 10.1007/s10571-026-01665-2.

## Introduction

Meningiomas are the most common primary tumors of the central nervous system (CNS), typically arising from meningothelial cells and considered benign and slow growing (Ogasawara et al. 2021). Despite their generally non-aggressive nature, their location within the dural membranes can lead to significant clinical symptoms depending on anatomical site (Ogasawara et al. 2021; Huntoon et al. 2020). They have a prevalence of 0.52% of the general population. They are more common in women, with risk factors including ionizing radiation, obesity, hormonal alterations, and pesticide exposure (Nakasu et al. 2021). Histologically, meningiomas account for 37.6% of primary CNS tumors and 53.3% of benign ones. Incidence rises with age, peaking in individuals over 40 years (Ostrom et al. 2021).

The World Health Organization (WHO) classifies meningiomas into three grades: grade 1 (benign), grade 2 (atypical), and grade 3 (malignant), based on distinct histological variants and biological behaviors (Wang et al. 2020a; Cohen-Inbar 2019; Lee et al. 2019). Grade 1 tumors represent about 80% of cases and have a more favorable prognosis compared to grades 2 and 3 (Sahm et al. 2017).

MicroRNAs (miRNAs), small non-coding RNAs that regulate gene expression post-transcriptionally by binding to mRNA targets, have emerged as essential players in tumor biology (Beylerli et al. 2022). Studies have demonstrated their involvement in key processes such as tumor initiation, proliferation, invasion, apoptosis, and recurrence in meningiomas (Galani et al. 2017; Werner et al. 2017). For instance, Carneiro et al. (2021) reported significantly elevated levels of miR-181d in meningioma tissues and plasma, with the highest expression in grade 3 tumors, suggesting a role in tumor aggressiveness (Carneiro et al. 2021).

Evaluating the differential expression of miRNAs between benign and malignant meningiomas may provide insights into their functional roles in tumor progression. Although multiple studies have explored miRNA expression in meningioma, consistent and clinically applicable signatures have yet to be established. This systematic review aims to assess the diagnostic, prognostic, and recurrence-related roles of miRNAs in meningioma, identifying expression patterns that may contribute to improved risk stratification and clinical decision-making.

## Methods

We conducted this review in accordance with the recommendations of the Cochrane Collaboration (Higgins 2011). We also followed the PRISMA statement guidelines (Liberati et al. 2009).

### Eligibility Criteria

*Study Design*: This systematic review included cohort, case-control studies and cross-sectional studies.

*Participants:*Included studies involved individuals diagnosed with meningiomas who received standard clinical interventions such as craniotomy or radio surgery, in accordance with local guidelines and included a minimum follow-up period of one year.

*Cases:* Participants diagnosed with meningioma according to the diagnostic guidelines of the country where the study was conducted. The differential expression of microRNAs was measured in a biological sample (blood, serum, plasma, cerebrospinal fluid, or tumor tissue).

*Controls: *Participants without clinical or imaging evidence of meningioma or any other type of tumor in the central nervous system, according to the guidelines of the country of origin of the study. Participants from whom a comparable sample was taken to evaluate the differential expression of microRNAs are also defined as controls. For tissue samples, participants who underwent surgery for another medical reason (e.g., stroke) or post-mortem within 24 hours of death are included.

*Primary Outcome: *The main outcome was the change in miRNA expression in individuals with meningiomas compared to those without the tumor, as well as variations in expression following clinical treatment. These findings were analyzed in relation to recurrence risk and stratified by histological grade according to the WHO classification.

*Follow-Up*: All studies were required to report outcomes with a follow-up of at least one year.

*Exclusion Criteria: *Studies were excluded if they included participants with non-meningioma tumors, tumors of metastatic origin, lacking surgical intervention or miRNA assessment, or were based on animal or in vitro models. 

### Databases and Search Strategy

We conducted searches on PubMed, Scopus, Web of Science, and Google Scholar from inception to the present. To ensure literature saturation, reference lists of relevant articles were screened, including grey literature such as conference abstracts, theses, OpenGrey, and ClinicalTrials.gov. In cases of missing or unclear data, study authors were contacted via email. No restrictions were applied regarding language or publication format (online Appendix 1).

### Risk of Bias

The risk of bias was assessed for each study using the Newcastle-Ottawa Quality Assessment Scale for case-control, cohort studies and cross-sectional studies, evaluating selection, comparability, and exposure.

### Synthesis of Results

A meta-analysis was not performed due to substantial heterogeneity among the included studies.

### Additional Analysis

*Target Pathway Analysis*: MicroRNAs (miRNAs) are key regulators of gene expression at the posttranscriptional level, as they simultaneously modulate multiple biological pathways. A single miRNA can regulate several target genes, and different miRNAs can converge on the same pathway, forming complex regulatory networks. This functional redundancy limits their individual specificity, so their potential clinical application is mainly considered complementary to other biomarkers. In this review, an enrichment analysis of metabolic pathways was performed for miRNAs that showed statistically significant differences in expression, according to the included studies, considering only those reported recurrently in at least two independent studies. miRNAs were grouped according to the origin of the biological sample: serum, tumor tissue, and cerebrospinal fluid and bioinformatic analysis were performed using the online tools miRPath v3.0 and TargetScan (Tastsoglou et al. 2023). This analysis allowed us to explore the involvement of these miRNAs in oncogenic pathways, particularly those related to cell cycle regulation, which are strongly implicated in recurrence and tumor progression in meningiomas.

## Results

### Study Selection

**Fig. 1:**
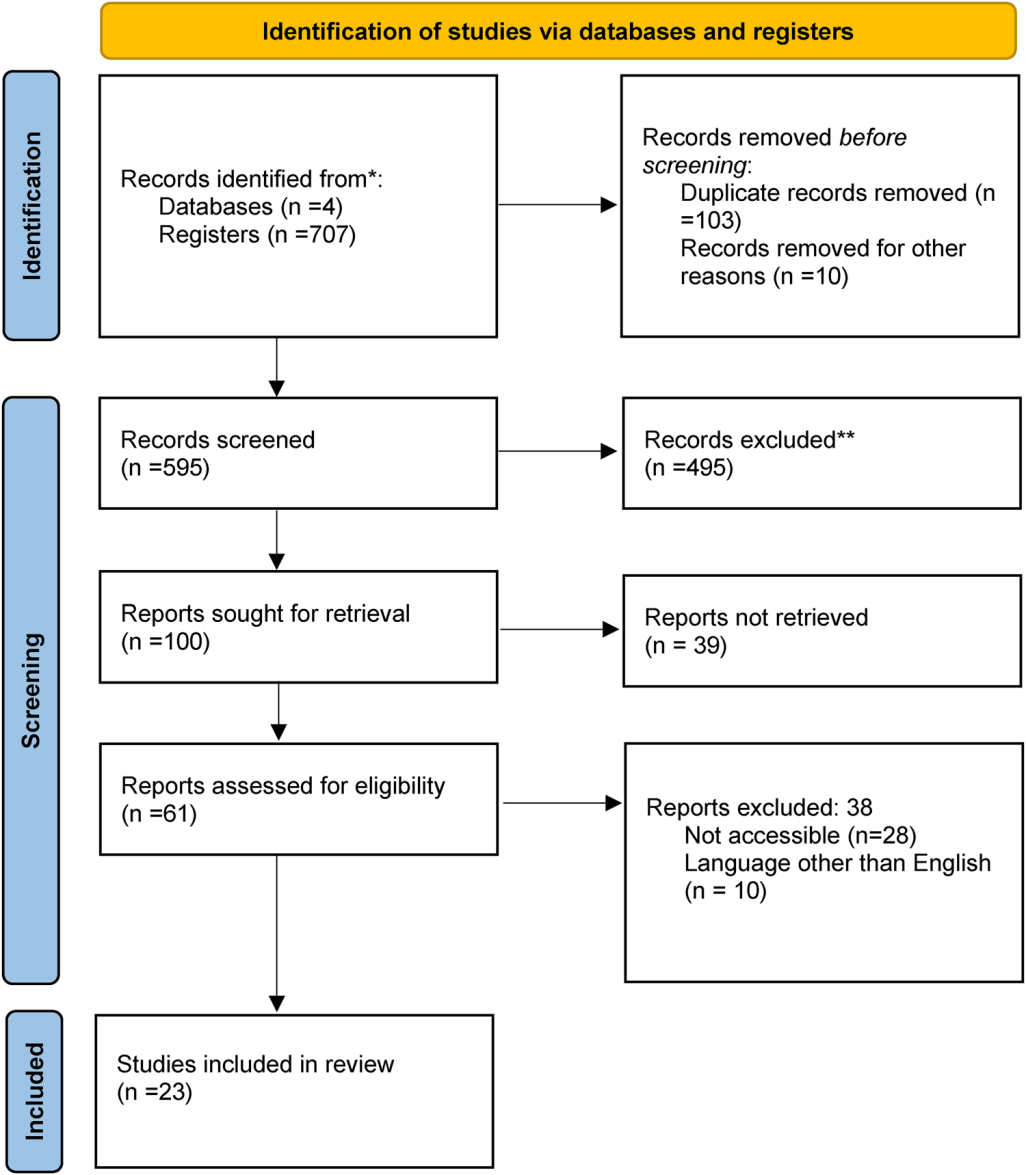
Flow diagram of included studies. *Consider, if feasible to do so, reporting the number of records identified from each database or register searched (rather than the total number across all databases/registers). **If automation tools were used, indicate how many records were excluded by a human and how many were excluded by automation tools. Source: Page MJ, et al. BMJ 2021;372:n71. doi: 10.1136/bmj.n71. This work is licensed under CC BY 4.0. To view a copy of this license, visit https://creativecommons.org/licenses/by/4.0/

Database searches yielded 707 studies. After removing duplicates, 595 records remained. In the initial review by title and abstract, considering the inclusion and exclusion criteria, 100 articles remained. In the full-text review, 61 studies with the necessary information remained. Finally, 49 studies remained for full review. Applying the eligibility criteria, 23 studies were included for data extraction and qualitative analysis. Only cohort and case-control studies were found at the time of the search, and therefore these were the only ones included (Fig. 1).

### Characteristics of the Included Studies

The 23 studies selected for this review included 8 cohort and 15 case-control designs, published between 2013 and 2024. They were conducted across Europe (11 studies), Asia (9), and the Americas (3), with a combined total of 1,615 participants. Among these, 1,461 had confirmed meningioma diagnoses: 658 with WHO grade 1, 278 with grade 2, and 329 with grade 3 tumors. The control group comprised 539 individuals, with samples obtained from peripheral blood or arachnoid tissue during unrelated cranial procedures (e.g., stroke surgeries) or post-mortem. The average age across studies was 57.89 ± 4.18 years.

Most studies focused on tumor tissue samples, though several—including those by Carneiro (2021), Zhi (2016), Abdelrahman (2022), Aran (2024), Kılıç (2022), Imura (2024), and Urbschat (2023)—analyzed serum. Kopkova (2019) uniquely studied cerebrospinal fluid. qRT-PCR was the most common analytical method, while others used digital RT-PCR, immunohistochemistry, or sequencing for miRNA profiling (Table 1).

**Table 1:** Characteristics of included studies

References	Settings	Country	Study Population (*n*)	Study desing	Age (x̅ + SD)	*S*ample evaluated	Confirmatory diagnostic method	Participants with meningioma (*n*)	Number of cases by histologic differentiation of the tumor		
									WHO 1	WHO 2	WHO 3
Abdelrahman (2022)	Royal Preston Hospital	United Kingdom	127	Case and control	59.2 ± 2,3	Serum	Tomography and MIR	74	25	25	24
Chen (2022)	Department of Neurosurgery, the Second Hospital of Hebei Medical University	China	50	Cohorts	56.82 ± 11.58	Tissue	Tomography and MIR	50	30	15	20
Rosa (2020)	University Hospital of the Faculty of Medicine of Ribeirão Preto of the University Sao Paulo.	Brazil	50	Case and control	57,2 ± 2,7	Tissue and serum	Tomography and MIR	40	16	16	8
Hergalant (2023)	Department of Pathology of the University Hospital of Nancy	France	80	Case and control	63.25 ± 7.25	Tissue	Tomography and MIR	60	32	28	–
Baran (2023)	İstanbul Training and Research Hospital, Neurosurgery	Turkey	30	Case and control	57.89 ± 16	Tissue	Tomography and MIR	30	–	–	–
Zhang (2020)	Neurosurgery department at the Beijing Tiantan Hospital.	China	55	Case and control	52	Tissue	Tomography and MIR	55	–	–	55
Wang (2015)	Hospital of PLA, Department of Neurosurgery, 15 Dongjiangwan Road, Shanghai	China	107	Cohorts	50.98 ± 7,43	Tissue	Tomography and MIR	107	52	26	25
Slavik (2020)	Institute of Molecular and Translational Medicine, Faculty of Medicine and Dentistry, Palacky University and University Hospital Olomouc	Czech Republic	172	Cohorts	52.5 ± 8,51	Tissue	Tomography and MIR	172	52	26	94
Aran (2024)	Instituto Estadual do Cérebro Paulo Niemeyer	Brazil	34	Case and controls	55.75 ± 16.74	Serum	Tomography and MIR	28	19	9	–
Katar (2017)	Istanbul Research and Training hospital	Turkey	50	Cohorts	55.96 ± 11.35	Tissue	Tomography and MIR	50	25	15	5
Li (2015)	Laiwu City People’s Hospital	China	117	Case and controls	58,3 ± 15,2	Tissue, serum and cerebrospinal fluid	Tomography and MIR	69	18	21	30
Ludwig (2015)	Institute of Pathology Medical School, Saarland University, Homburg/Saar	Germany	55	Cohorts	58,3 ± 6,39	Tissue	Tomography and MIR	55	33	12	10
Wang (2020)	Department of Infectious Diseases, Xiangya Hospital, Central South University, Changsha, China.	China	106	Case and controls	65,2 ± 2,65	Tissue	–	96	–	–	–
Kılıç (2022)	Medicana International Ankara Hospital Research	Turkey	30	Case and controls	55,3 ± 2,5	Tissue	Tomography and MIR	15	10	3	2
Imura (2024)	Department of Neurosurgery, Hiroshima University Hospital	Japan	9	Cohorts	58,3 ± 5,26	Serum	Tomography and MIR	9	5	4	–
Kopkova (2019)	Department of Neurosurgery, University Hospital Brno	Czech Republic	95	Case and controls	58,3 ± 2,89	Cerebrospinal fluid	Tomography and MIR	55	–	–	–
Urbschat (2023)	Department of Neurosurgery, Saarland University	Germany	63	Case and controls	61,7 ± 13,85	Tissue and serum	Tomography and MIR	43	37	7	1
Zhi (2016)	Second Affiliated Hospital of Soochow University	China	460	Case and controls	57.3 ± 12.1	Serum	Tomography and MIR	230	175	23	32
Zhi (2013)	Department of Neurosurgery, Third Affiliated Hospital of Soochow University.	China	145	Case and controls	54,06 ± 11,01	Tissue	Tomography and MIR	110	60	18	12
Galani (2015)	Department of Anatomy-Histology-Embryology, Epirus	Greece	17	Cohorts	60.3 ± 16.3	Tissue	Tomography and MIR	17	11	4	2
El-Gewely (2016)	University Hospital of North Norway, Tromsø	Norway	23	Case and controls	61,3 ± 11,96	Tissue	Tomography and MIR	18	12	3	–
Carneiro (2021)	Surgery and Anatomy, University of São Paulo, Ribeirão Preto Medical School, Ribeirão Preto, BRA	Brazil	46	Case and controls	–	Tissue and serum	Tomography and MIR	40	16	16	8
Duba (2024)	Department of Neurosurgery, University Hospital Brno, Brno	Czech Republic	38	Cohorts	61,7 (48,8–70,0)	Tissue	Tomography and MIR	38	30	7	1

References	Settings	Country	Controls population		*P *value	microRNAs determinated	Regulated to the upside	Regulated downwards	Laboratory measurement
			*n*	Characteristic of the population					
Abdelrahman (2022)	Royal Preston Hospital	United Kingdom	53	Blood donors	< 0,05	miR-497and miR-219	miR-219	miR-497	qRT-PCR
Chen (2022)	Department of Neurosurgery, the Second Hospital of Hebei Medical University	China	–	–	< 0,05	miR-153-5p	–	miR-153-5p	qRT-PCR, immunohistochemistry and western blot
Rosa (2020)	University Hospital of the Faculty of Medicine of Ribeirão Preto of the University Sao Paulo.	Brazil	10	Arachnoid obtained from aneurysm surgeries	< 0,05	miR-34a, miR-143, miR-145 and miR-335	–	miR-34a and miR-145	qRT-PCR
Hergalant (2023)	Department of Pathology of the University Hospital of Nancy	France	20	Cadaver dura mater 10 and surgical dura mater 10	< 0,05	miR-16 and miR-519	miR-16 and miR-519	–	qRT-PCR and western blot
Baran (2023)	İstanbul Training and Research Hospital, Neurosurgery	Turkey	6	Dura mater membranes from cadavers belonging to individuals who died from extracranial causes.	< 0,05	miR-451 and miR-885	miR-451	miR-885	qRT-PCR
Zhang (2020)	Neurosurgery department at the Beijing Tiantan Hospital.	China	43	Atypical radiosensitive meningiomas	< 0,05	miR-4286, miR-4695-5p, miR-6732-5p, miR-6855-5p, miR-7977, miR-6765-3p, miR-6787-5p, miR-1275, miR-30c-1-3p, miR-4449, miR-4539, miR-4684-3p, miR-6129, miR-6891-5p	miR-1275, miR-30c-1-3p, miR-4449, miR-4539, miR-4684-3p, miR-6129, miR-6891-5p	miR-4286, miR-4695-5p, miR-6732-5p, miR-6855-5p, miR-7977, miR-6765-3p, miR-6787-5p	qRT-PCR
Wang (2015)	Hospital of PLA, Department of Neurosurgery, 15 Dongjiangwan Road, Shanghai	China	–	–	< 0,05	miR-224	miR-224	–	qRT-PCR and western blot
Slavik (2020)	Institute of Molecular and Translational Medicine, Faculty of Medicine and Dentistry, Palacky University and University Hospital Olomouc	Czech Republic	–	–	< 0,05	hsa-miR-15a-5p, hsa-miR-19b-3p, hsa-miR-30e-5p, hsa-miR-107, hsa-miR-146a-5p, hsa-miR-320c, and hsa-miR-331-3p	miR-331-3p	–	qRT-PCR
Aran (2024)	Instituto Estadual do Cérebro Paulo Niemeyer	Brazil	34	Blood donors	< 0,05	miR-21	miR-21	–	Digital RT-PCR
Katar (2017)	Istanbul Research and Training hospital	Turkey	.	.	< 0,05	miR-21, miR-107, miR-137 and miR-29b	miR-21, miR-107, miR-137 and miR-29b	–	qRT-PCR
Li (2015)	Laiwu City People’s Hospital	China	48	Normal tissues were collected as controls and stored in liquid nitrogen. Fasting peripheral blood was also collected and stored in EDTA tubes at −20 °C, while 2 ml of cerebrospinal fluid were collected, centrifuged, and stored at −20 °C	< 0,05	miR-18a	miR-18a		qRT-PCR
Ludwig (2015)	Institute of Pathology Medical School, Saarland University, Homburg/Saar	Germany	–	–	< 0,05	miR-136, −195, −222, −497, −376c, and − 34a	miR-34a	–	qRT-PCR
Wang (2020)	Department of Infectious Diseases, Xiangya Hospital, Central South University, Changsha, China.	China	9	–	< 0,05	miR-98-5p	miR-98-5p	–	qRT-PCR
Kılıç (2022)	Medicana International Ankara Hospital Research	Turkey	15	Arachnoid obtained from aneurysm surgeries	< 0,05	miR-23a/b y miR-21	miR-23a/b y miR-21	–	qRT-PCR
Imura (2024)	Department of Neurosurgery, Hiroshima University Hospital	Japan	–	–	< 0,05	hsa-miR-664b, hsa-miR-7706, hsa-miR-590, hsa-miR-6513, hsa-miR-193a	hsa-miR-664b, hsa-miR-7706, hsa-miR-590, hsa-miR-6513	hsa-miR-193a	qRT-PCR
Kopkova (2019)	Department of Neurosurgery, University Hospital Brno	Czech Republic	40	Patients with hydrocephalus	< 0,05	miR-196a-5p, miR-4306, miR-10a-5p, miR-4791, miR-30c-5p	miR-196a-5p, miR-4306	–	qRT-PCR
Urbschat (2023)	Department of Neurosurgery, Saarland University	Germany	20	Completely healthy controls	< 0,05	miR21; miR34a; miR200a and miR409	miR-200a and miR 409	miR-200a in blood	qRT-PCR
Zhi (2016)	Second Affiliated Hospital of Soochow University	China	230	Completely healthy controls	< 0,05	miR-106a-5p, miR-219-5p, miR-375 y miR-409-3p, miR-197 y miR224	miR-106a-5p, miR-219-5p, miR-375 y miR-409-3p	.	qRT-PCR
Zhi (2013)	Department of Neurosurgery, Third Affiliated Hospital of Soochow University.	China	45	Completely healthy controls	< 0,05	miR-17-5p, miR-22-3p, miR-24-3p, miR-26b5p, miR-27a-3p, miR-27b-3p, miR-96-5p, miR-146a-5p, miR155-5p, miR-186-5p, miR-190a y miR-199a	miR-17-5p, miR-22-3p, miR-24-3p, miR-26b5p, miR-27a-3p, miR-27b-3p, miR-96-5p, miR-146a-5p, miR155-5p, miR-186-5p, miR-190a y miR-199a	miR-29c-3p y miR-219-5p	qRT-PCR
Galani (2015)	Department of Anatomy-Histology-Embryology, Epirus	Greece	–	–	< 0,05	miR-21	miR-21	–	
El-Gewely (2016)	University Hospital of North Norway, Tromsø	Norway	–	–	< 0,05	miR-130a, miR-143, miR-148b, miR-152, miR-193b, miR-199a-5p, miR-21, miR-218, miR-26b, miR-34a, miR-342-3p, miR-376c, miR-424, miR-451, miR-574-3p, miR-99a	miR-34a, miR-199 and mR26b	–	qRT-PCR and SOLiD sequencing (third generation)
Carneiro (2021)	Surgery and Anatomy, University of São Paulo, Ribeirão Preto Medical School, Ribeirão Preto, BRA	Brazil	–	–	< 0,05	miR-181d, miR-181c and miR-130a	miR-181d	El miR-181c and miR-130a	qRT-PCR
Duba (2024)	Department of Neurosurgery, University Hospital Brno, Brno	Czech Republic	–	–	< 0,05	miR-124-3p, miR-130a-5p, miR-675-3p, miR-130a-3p, miR-675-5p, miR-17-5p, miR-671-3p, miR-3126-3p, miR-6842-3p, miR-340-3p, miR-34c-3p, miR-30b-5p, miR-2114-3p, miR-144-3p, miR-31-5p, miR-30e-5p, miR-34c-5p, miR-32-3p, miR-181a-5p, miR-1911-5p, miR-425-5p, miR-625-3p, miR-92b-3p, miR-6726-3p, miR-10527-5p, miR-1299, miR-2277-5p, miR-6511b-3p, miR-2114-5p, miR-625-5p, miR-6516-5p, miR-19a-3p, miR-449a, miR-491-5p, miR-1298-5p, miR-34b-3p, miR-374a-3p, miR-1843, miR-1264, miR-181b-5p, miR-3160-3p, miR-548i, miR-548o-3p, miR-548ba, miR-590-5p, miR-100-5p, miR-30e-3p, miR-501-3p, miR-324-5p, miR-483-5p, miR-151a-5p, miR-3129-5p, miR-548 h-3p, miR-504-5p, miR-186-3p, miR-34b-5p, miR-378a-3p, miR-339-3p, miR-361-5p, miR-592, miR-486-3p, miR-21-5p, miR-1179, miR-671-5p, miR-132-3p, miR-149-5p, miR-549a-5p, miR-203b-3p, miR-423-3p, miR-628-5p, miR-5010-3p, miR-3117-3p.	miR-124-3p, miR-675-3p, miR-675-5p, miR-671-3p, miR-6842-3p, miR-34c-3p, miR-2114-3p, miR-31-5p, miR-34c-5p, miR-181a-5p, miR-625-3p, miR-6726-3p, miR-1299, miR-6511b-3p, miR-625-5p, miR-6516-5p, miR-449a, miR-1298-5p, miR-34b-3p, miR-1843, miR-181b-5p, miR-548i, miR-548ba, miR-100-5p, miR-501-3p, miR-483-5p, miR-3129-5p, miR-504-5p, miR-34b-5p, miR-378a-3p, miR-339-3p, miR-361-5p, miR-592, miR-486-3p, miR-21-5p, miR-1179, miR-671-5p, miR-132-3p, miR-149-5p, miR-549a-5p, miR-203b-3p, miR-423-3p, miR-628-5p, miR-5010-3p y miR-3117-3p.	miR-130a-5p, miR-130a-3p, miR-17-5p, miR-3126-3p, miR-340-3p, miR-30b-5p, miR-144-3p, miR-30e-5p, miR-186-3p, miR-32-3p, miR-1911-5p, miR-625-3p, miR-6726-3p, miR-1299, miR-6511b-3p, miR-625-5p, miR-19a-3p, dejar-7d-3p, miR-1298-5p, miR-374a-3p, miR-1264, miR-3160-3p, miR-548o-3p, miR-590-5p, miR-30e-3p, miR-324-5p, miR-151a-5p y miR-548 h-3p.	NextSeq (second generation) sequencing

### Characteristics of Excluded Studies

Excluded articles did not meet the inclusion criteria or were review articles, database-based studies, or studies assessing the functional role of specific miRNAs in cellular processes.

### Association Between miRNAs and Meningioma

A total of 153 miRNAs were evaluated across 23 studies for their association with meningioma. Of these, 90 were upregulated, 45 downregulated, and 18 showed no significant differential expression. Although a wide range of miRNAs was reported, several were consistently identified across multiple independent studies, suggesting a stronger association with meningioma biology.

In tissue, the most frequently evaluated miRNA was miR-21. Several studies described an increase in its expression associated with histological grade or tumor aggressiveness (Katar et al. 2017a; Galani et al. 2015; Kılıç et al. 2022; Urbschat et al. 2023), although not all studies observed statistically significant differences between grades or in relation to recurrence (Galani et al. 2015; Urbschat et al. 2023).

A convergent pattern of progressive decrease with tumor grade was described for miR-153-5p, whose expression was significantly lower in higher-grade tumors and dural invasion (Chen et al. 2022). Similarly, miR-16 and miR-519 showed a consistent reduction in tumor tissue compared to controls, with no differences between grades or association with recurrence (Hergalant et al. 2023).

Regarding miR-18a, the results were discordant between studies. While significantly reduced levels were reported in invasive and non-invasive meningiomas compared to controls (Li et al. 2015), other studies observed a progressive increase with histological grade, with approximately double levels in grade III tumors (Carneiro et al. 2021).

Several miRNAs showed repeated associations with tumor grade, recurrence, or survival. miR-224-5p showed a progressive increase with histological grade and was associated with higher recurrence and lower survival (Wang et al. 2015a). miR-331-5p, miR-15a-5p, and miR-146a-5p were consistently associated with recurrence, with independent prognostic value for miR-331-5p (Slavik et al. 2020a, b). Similarly, decreased miR-29c-3p and miR-219-5p were associated with higher recurrence rates, while miR-190a was associated with lower recurrence-free survival (Zhi et al. 2013).

Other studies identified specific profiles without subsequent replication, including overexpression of miR-451 without association with grade(Baran et al. 2023), miRNA profiles associated with radioresistance (Zhang et al. 2020), differences according to histological subtype (Ludwig et al. 2015), differential expression in rigid tumors (Duba et al. 2024), and associations with TF-miRNA networks without a pattern of progression by grade (Wang et al. 2020b). The expression of miR-34a and miR-145 was reduced in grade II without a consistent progressive pattern (Rosa et al. 2020), and miR-191 showed differential expression without clear clinical association (El-Gewely et al. 2016).

In serum, the evidence was more limited, but some recurring patterns were identified. A profile characterized by increased miR-106a-5p, miR-219-5p, miR-375, and miR-409-3p, along with decreased miR-197 and miR-224, was described and validated in independent cohorts (Zhi et al. 2016).

Other studies reported an overall decrease in multiple serum miRNAs and a specific increase in miR-193a (Imura et al. 2024). miR-18a showed reduced levels in both invasive and non-invasive meningiomas compared to controls (Li et al. 2015), while miR-18d increased progressively with tumor grade (Carneiro et al. 2021). No significant differences were observed between grades for miR-34a, miR-145, miR-143, and miR-335 (Rosa et al. 2020), nor were there any differences compared to healthy controls for miR-21 (Aran et al. 2024). miR-200a showed differences by sex, with no association with recurrence (Urbschat et al. 2023).

In CSF, only two studies evaluated miRNA. Significant expression of miR-196a-5p, miR-10a-5p, miR-196b-5p, miR-199b-3p, miR-140-5p, and miR-10b-5p was described, proposed for the evaluation of meningiomas (Kopkova et al. 2019). Consistently, miR-18a showed significantly reduced levels in CSF from invasive and non-invasive meningiomas, with a greater decrease in invasive cases (Li et al. 2015) (Table 2).

**Table 2:** miRNAs identified with high and low gene expression

Sample evaluated	References	miRNAS	Principal findings
Tissue	Chen (2022)	hsa- miR-153-5p	The expression of has- miR-153-5p is significantly decreased in meningiomas, with progressively lower levels in tumors of higher histological grade and in those with dural invasion, independently associated with greater tumor aggressiveness.
	Hergalanat (2023)	hsa-miR-16 and hsa-miR-519	were found to be significantly reduced in meningioma tissues compared to controls, with no differences between grades I and II, and no association with tumor recurrence, and therefore do not correlate with the progression or prognosis of meningioma.
	Baran (2023)	hsa-miR-885 and hsa-miR-451	miRNA-451 showed overexpression in meningiomas with diagnostic value compared to control tissues, while miRNA-885 showed no differences; neither was associated with tumor grade.
	Zhang (2020)	hsa- miR-4286, hsa-miR-4695-5p, hsa-miR-6732-5p, hsa-miR-6855-5p, hsa-miR-7977, hsa-miR-6765-3p, hsa-miR-6787-5p	Its expression is associated with radioresistant atypical meningiomas.
	Katar (2017)	hsa-miR-21, hsa-miR-107	has-miR-21 showed a progressive increase with meningioma grade, associating with greater tumor aggressiveness, while has-miR-107 showed a decrease in higher-grade tumors, suggesting an inverse association with malignancy.
	Ludwig (2015)	hsa-miR-222, hsa-miR-195, hsa-miR-497	Increased expression in fibroblasts and transitional cells
		has-miR-18a, has-miR-18b	Increased expression in the meningothelial subtype
	Kılıç (2022)	hsa-miR-23b, hsa-miR-21, hsa-miR-23a	High expression in low-grade tumors
	Urbschat (2023)	hsa-miR-200a, hsa-miR-409	MicroRNA-200a was found to be decreased in recurrent meningiomas, regardless of WHO grade. MicroRNA-409 was associated with larger tumor volume and younger patient age. No differences were observed for microRNA-21 or microRNA-34a, and associations were identified between microRNA-34a and microRNA-200a with deletions of chromosome 1p, as well as between microRNA-409 and alterations of chromosome 14.
	Wang (2015)	hsa- miR-224-5p	showed a progressive increase with the histological grade of meningioma, associated with higher tumor recurrence and lower survival, supporting its oncogenic role in tumor progression and aggressiveness.
	Slavik (2020)	hsa-miR-331-5p, hsa-miR-15a-5p, hsa-miR-146a-5p	Were significantly associated with time to recurrence, with miR-331-3p being the only microRNA with independent prognostic value in the multivariate analysis.
	Zhi (2013)	hsa-miR-17-5p, hsa- miR-22-3p, hsa-miR-24-3p, hsa-miR-26b-5p, hsa-miR-27a-3p, hsa-miR-27b-3p, hsa-miR-96-5p, hsa-miR-146a-5p, hsa-miR-155-5p, hsa-miR-186-5p, hsa-miR-190a, hsamiR-199a	Overexpressed in meningioma tissue, regardless of grade
		hsa-miR-29c-3p, hsa- miR-219-5p	decreased expression in meningioma tissue
		hsa-miR-190a, hsa-miR-29c-3p, hsa-miR-219-5p	hsa-miR-190a patients had lower recurrence-free survival, while those with low expression of hsa-miR-29c-3p and hsa-miR-219-5p showed a significantly higher recurrence rate. These findings were consistent in both the training and validation sets.
	Galani (2015)	hsa-miR-21	Higher expression in grade II tumors, without statistical significance.
	Duba (2024)	hsa-miR-124-3p, hsa-miR-675-p, hsa-miR-675-5p, hsa-miR-130a-3p y hsa-miR-130a-5p	Documented significant expression of hsa-miR-124-3p, hsa-miR-675-p and hsamiR-675-5p in stiff tumors, whereas hsa-miR-130a-3p and hsa-miR-130a-5p showed down-regulation.
	El-Gewely (2016)	hsa-miR-191, hsa-miR16	Differential expression was reported in meningiomas.
	Rosa (2020)	hsa-miR-34a hsa-miR-145	They showed significantly reduced expression in grade II meningiomas compared to grades I and III; however, they did not show a progressive pattern related to increased tumor grade, and therefore do not correlate consistently with meningioma progression.
	Li (2015)	hsa-miR-18a	was significantly lower in invasive meningiomas compared to controls and non-invasive meningiomas, and was also found to be reduced in non-invasive meningiomas compared to control tissues.
	Duba (2024)	hsa-miR-31-5p, hsa-miR-34b-5p, hsa-miR-483-5p	Differential expression in rigid type I meningiomas
	Carneiro (2021)	hsa-miR-18a	increased significantly with tumor grade, being approximately double in grade III meningiomas compared to grade I.
	Wang (2020)	hsa-miR-574-5p, hsa-miR-26b-5p, hsa-miR-335-5p hsa-miR-98-5p	They were identified expressed in meningiomas, associated with TF-miRNA correlation networks and 3 key genes.
Serum	Abdelrahman (2022)	hsa-miR-497, hsa-miR-129	Has-miR-497 is inversely associated with meningioma aggressiveness, with elevated levels in benign tumors and a progressive decrease in high grades, while has-miR-219 shows the opposite pattern, with increased levels in more aggressive meningiomas.
	Imura (2024)	hsa-miR-664b, hsa-miR-7706, hsa-miR-590, hsa-miR-6513,	Decrease in expression
		hsa-miR-193a	Increase in expression
	Zhi (2016)	hsa-miR-106a-5p, hsa-miR-219-5p, hsa-miR-375, hsa-miR-409-3p	This pattern of increase was consistent and validated in independent cohorts, supporting its usefulness as a serum marker associated with meningioma.
		hsa-miR-197, hsa-miR-224	This reduction was reproducible in the training and validation sets, confirming its involvement in the differential profile of meningioma.
	Rosa (2020)	hsa-miR-34a, hsa-miR-145, hsa-miR-143 and hsa-miR-335	No statistically significant differences were observed between the different tumor grades, as levels were similar regardless of histological grade, indicating that these microRNAs are not useful as biomarkers of tumor progression in meningiomas.
	Li (2015)	hsa-miR-18a	showed significantly reduced levels in invasive and non-invasive meningiomas compared to controls, with no differences between the two tumor subtypes.
	Urbschat (2023)	has-miR-200a	It was found to be overexpressed in the male cohort, but no difference was associated between primary and recurrent meningiomas.
	Carneiro (2021)	has-miR-18d	increased significantly with tumor grade, being approximately double in grade III meningiomas compared to grade I.
	Aran (2024)	hsa-miR-21	No significant differences were observed with healthy controls.
Cerebrospinal fluid	Kopkova (2019)	hsa-miR-196a-5p, hsa-miR-10a-5p, hsa-miR-196b-5p, has-miR-199b-3p, hsa-miR-140-5p, hsa-miR-10b-5p	Significant expression; potential for meningioma evaluation.
	Li (2015)	hsa-miR-18a	showed significantly reduced levels of invasive and non-invasive meningiomas compared to controls, with a greater decrease in invasive cases.

### Risk of Bias Analysis

Risk of bias was assessed using the Newcastle-Ottawa Scale (Peterson et al. 2011). Twelve of the 23 studies were classified as having low risk of bias, based on criteria evaluating selection, comparability, and case exposure. The studies by Duba (2024), Imura (2024), and Chen (2022) were considered to have intermediate risk due to inadequate cohort follow-up. Similarly, the studies by Zhi (2013, 2016), Urbschat (2023), Kopkova (2019), Li (2015), and Rosa (2020) were also classified as intermediate risk because their control groups were not community-based. Lastly, the studies by Kılıç (2022) and Wang (2020) were rated as high risk of bias due to insufficient representation, selection, and definition of control groups (Supplementary Figs. 1a, b and 2a, b).

### Heterogeneity

Heterogeneity among studies was a major limitation and the primary reason was that a meta-analysis was not performed. Variability arose from differences in sample type (tumor tissue, serum/plasma, cerebrospinal fluid), with several miRNAs showing compartment-specific expression. Additional heterogeneity was related to the analytical platforms used (qPCR, microarray, next-generation sequencing), which differ in sensitivity and normalization approaches. Moreover, clinical and population-related factors, including tumor grade distribution, outcome definitions, and geographic origin, further limited direct comparability across studies.

### Target Pathway Analysis of miRNAs

By grouping the microRNAs and applying the criteria established in the methodology, only five microRNAs were found to be overexpressed and reported by the studies with statistical significance, suggesting them as potential biomarkers. The miRPath analysis showed a statistically significant association with several pathways involved in oncogenic metabolic and immunological regulation and configuration. The main involvement of miR-146a was found in the NF Kappa Beta pathway and miR-21 in gap junctions.

**Fig. 2:**
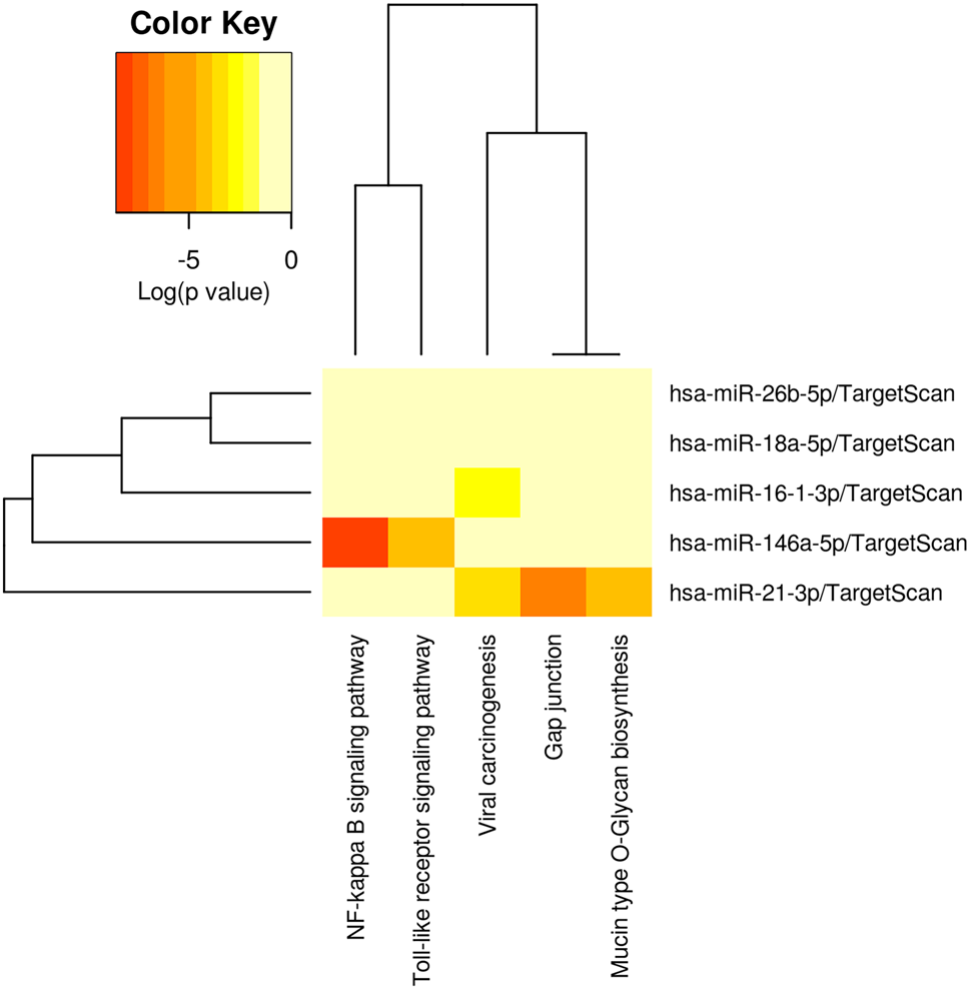
Heatmap and cluster patterns. microRNAs overexpressed in tumor tissue

A lesser involvement, but with a statistically significant value, was found for miR-16-3p in pathways associated with viral carcinogenesis (Fig. 2).

## Discussion

### Summary of Main Findings

This systematic review identified a set of miRNAs consistently associated with the grade, recurrence, and progression of meningioma disease, supporting their potential clinical utility as biomarkers. Among them, miR-21, miR-224-5p, miR-331-5p, and miR-29c-3p were most frequently associated with tumor recurrence and progression in independent studies. In addition, circulating miRNAs, such as miR-497 and miR-129, detected in serum and cerebrospinal fluid, demonstrated their diagnostic potential for tumor classification. Overall, these findings highlight the relevance of miRNA dysregulation in meningioma biology and support their role in disease stratification and monitoring.

### Contrast with Literature

The association between microRNAs (miRNAs) and meningioma remains in an exploratory phase, with current evidence being limited and heterogeneous (Chen et al. 2023). Our findings are consistent with previous meningioma-specific studies published over the last decade, which have repeatedly identified a limited subset of miRNAs associated with tumor grade, recurrence, and progression. miR-21 has been one of the most consistently reported miRNAs, showing increased expression in higher-grade and recurrent meningiomas across multiple tissue-based studies, supporting its role as a marker of tumor aggressiveness (Katar et al. 2017b). Similarly, altered expression of miR-34a and members of the miR-181 family (miR-181a, miR-181b, miR-181d) has been associated with tumor grade, histological subtype, and disease progression, with some studies providing functional or bioinformatic support for their involvement in meningioma biology (Eraky 2023). Several miRNAs associated with tumor recurrence, including miR-224-5p, miR-331-5p, miR-29c-3p, and miR-219-5p, have been reported in tissue-based comparisons of primary and recurrent meningiomas, although with heterogeneous expression patterns across studies. Notably, miR-224-5p has been linked to malignant progression through apoptotic pathway modulation, while miR-331-5p has demonstrated particularly strong and consistent associations with recurrence in multivariable analyses, highlighting its potential relevance within recurrence risk stratification panels (Wang et al. 2015b; Slavik et al. 2020b; Tekin et al. 2025).

In parallel, emerging studies on circulating miRNAs, particularly miR-497 and miR-219, suggest diagnostic potential for tumor grading in serum or plasma, but these findings remain limited by small sample sizes and methodological heterogeneity (Joshi et al. 2024). Overall, the repeated reporting of a small subset of miRNAs across independent cohorts strengthens their relevance in meningioma, while the variability observed for other candidates highlights the need for further validation in larger, standardized studies.

As for the nuclear factor kappa B (NF-κB) signaling pathway, there is evidence of its involvement in the pathogenesis and progression of meningiomas. NF-κB activation promotes processes such as cell proliferation, tumor invasion, resistance to apoptosis, and modulation of the tumor microenvironment (Tsitsikov et al. 2023; Maalim et al. 2024). In malignant meningiomas, the RACK1 protein has been shown to interact with the β subunit of casein kinase 2 (CSNK2B), preventing its degradation by ubiquitination. This allows CK2 to activate the NF-κB pathway, which increases the transcription of CDK4 and cyclin D3, promoting cell cycle progression and tumor malignancy (Tsitsikov et al. 2023).

In addition, the NF-κB pathway is involved in regulating NF2 gene methylation, which is essential in the tumorigenesis of benign meningiomas. Activation of NF-κB by proinflammatory cytokines such as IL-1β induces DNMT1 expression, which promotes NF2 promoter methylation and reduces merlin expression, facilitating tumor development (Wang et al. 2016). On the other hand, PD-L1 expression in meningiomas, associated with poorer prognosis and higher recurrence, correlates with NFKB2 expression, suggesting that the NF-κB pathway also contributes to tumor immune evasion, especially under hypoxic conditions (Karimi et al. 2020).

### Strengths and Limitations

This systematic review elucidates the expression profiles of miRNAs associated with meningioma and highlights their potential role as biomarkers for disease classification, prognosis, and follow-up. The findings provide a foundation for future research integrating miRNA profiling with molecular and cellular mechanisms, which may ultimately inform treatment decisions and surveillance strategies, contributing to reduced morbidity and mortality.

However, several limitations must be acknowledged. First, variability in sample types and control selection was evident across studies. Most control samples were not pathology-matched and were often chosen based on availability, reflecting the practical challenges of obtaining normal meningeal tissue. Second, the overall sample sizes of the included studies were limited, particularly for high-grade meningiomas, which restricts statistical power and the generalizability of findings. Third, most studies were exploratory in nature and lacked independent validation cohorts, limiting the robustness and reproducibility of reported miRNA signatures. Finally, substantial technical heterogeneity was introduced using different miRNA detection platforms and data normalization procedures, further complicating cross-study comparisons.

## Conclusion

The miRNAs, particularly miR-21, miR-224-5p, miR-331-5p, miR-29c-3p, and circulating miR-497, were recurrently associated with meningioma grade, recurrence, and disease progression. Despite methodological heterogeneity and predominantly exploration study designs, the consistency of these findings across independent cohorts supports their potential relevance as biomarkers. Bioinformatics analyses indicate convergence in metabolic and survival pathways, highlighting the miRNA-metabolism axis as a promising avenue for future research. However, larger, standardized studies with independent validation are required before these biomarkers can be applied in clinical practice.

## Supplementary Information

Below is the link to the electronic supplementary material.

Supplementary material 1 (DOCX 6.8 kb)

Supplementary material 2 (DOCX 20.2 kb)

Supplementary material 3 (TIF 75862.1 kb)—**A** Risk of bias assessment within the studies. **B **Risk of bias assessment across the studies cases and controls

Supplementary material 4 (TIF 2738 kb)—**A** Risk of bias assessment within the studies. **B** Risk of bias assessment across the studies Cohorts

## Data Availability

No datasets were generated or analysed during the current study.
