# Supplementary material for: Association Between miRNAs and the Diagnosis, Prognosis, and Recurrence of Patients with Meningioma: A Systematic Review
Source: Cell Mol Neurobiol. 2026 Feb 7;46:47. doi: 10.1007/s10571-026-01665-2 (PMC12923656; doi:10.1007/s10571-026-01665-2)
Supplement: Supplementary file 1 — Supplementary material 1 (DOCX 6.8 kb) [file 10571_2026_1665_MOESM1_ESM.docx]

**Search Strategy:**

**Pubmed:**

((Meningioma) OR (Meningioma, familial) ) AND (microRNAs)

**Scopus**:

TITLE-ABS-KEY ("meningioma") AND TITLE-ABS-KEY (microRNAs) OR TITLE-ABS-KEY (microrna) OR TITLE-ABS-KEY (RNA,micro) AND TITLE-ABS-KEY ( survival) OR TITLE-ABS-KEY (recurrence) OR TITLE-ABS-KEY (prognosis) ):

**Web Science:**

((ALL=(meningioma)) OR ALL=(meningioma,familial)) AND ALL=(microRNAs):

**Google Scholar:**

(("intracranial meningioma" OR "meningioma") AND ("microRNA" OR "microRNAs")) AND ("overall survival" OR "disease-free survival" OR "recurrence risk") AND ("expression profiling" OR "qPCR" OR "RNA sequencing") AND ("prognostic biomarkers" OR "therapeutic target" OR "diagnostic significance") AND ("tumor progression" OR "molecular pathways" OR "clinic)
