# Supplementary figures and images for: Association Between miRNAs and the Diagnosis, Prognosis, and Recurrence of Patients with Meningioma: A Systematic Review
Source: Cell Mol Neurobiol. 2026 Feb 7;46:47. doi: 10.1007/s10571-026-01665-2 (PMC12923656; doi:10.1007/s10571-026-01665-2)

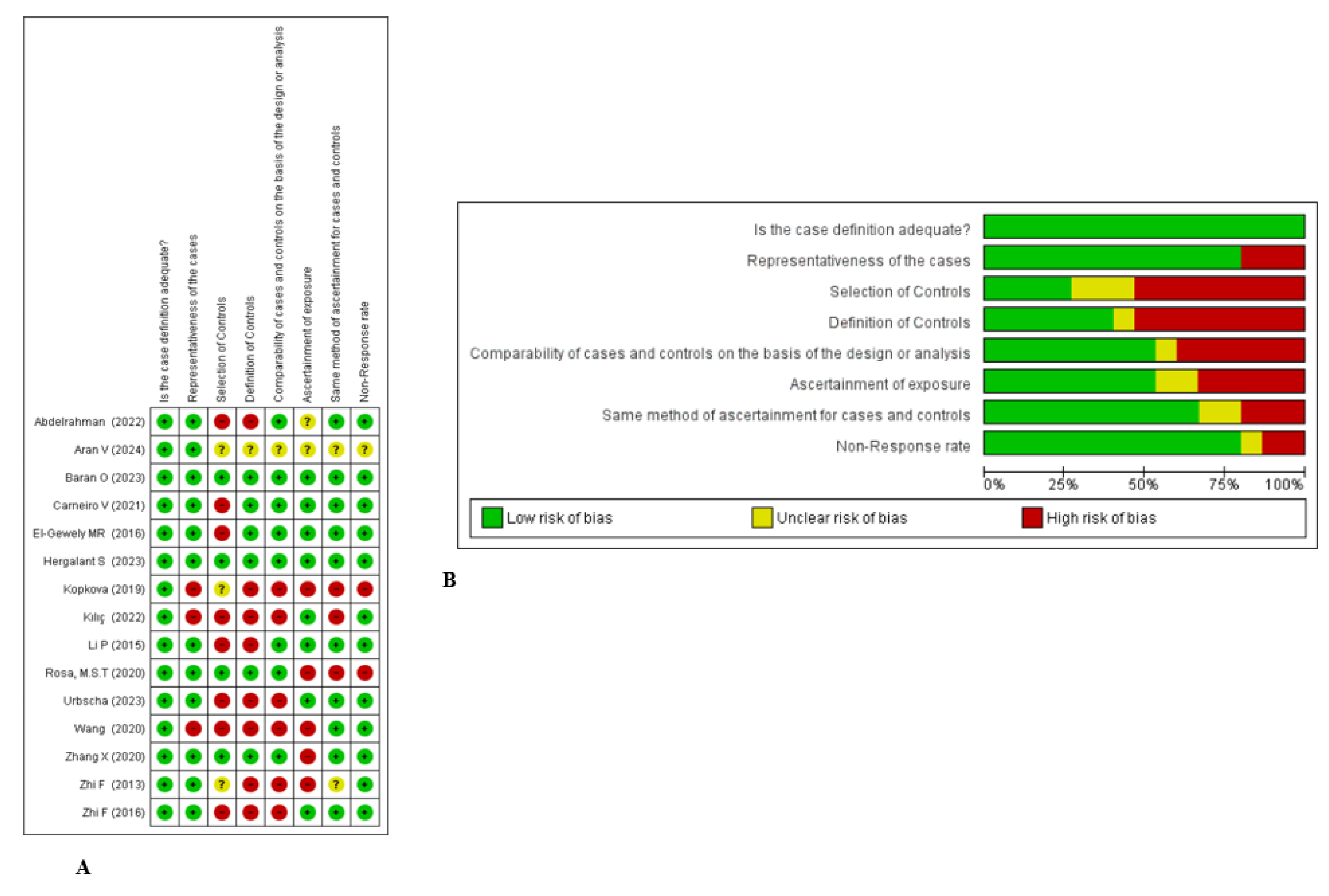

Supplement: Supplementary file 4 — Supplementary material 4 (TIF 2738 kb)—A Risk of bias assessment within the studies. B Risk of bias assessment across the studies Cohorts [file 10571_2026_1665_MOESM4_ESM.jpg]
